# Fit-for-Future: Lessons Learned from the COVID-19 Pandemic in Primary Extracorporeal Membrane Oxygenation (ECMO) Transports of Acute Respiratory Distress Syndrome (ARDS) Patients

**DOI:** 10.3390/jcm13185391

**Published:** 2024-09-12

**Authors:** Stefan Muenster, Silvia Schumacher, Mathias Schmandt, Stefan Kreyer, Jens Martin Poth, Christian Putensen, Jens-Christian Schewe, Stefan Felix Ehrentraut

**Affiliations:** 1 Department of Anesthesiology and Intensive Care Medicine, University Hospital Bonn, 53127 Bonn, Germany; silvia.schumacher@ukbonn.de (S.S.); mathias.schmandt@ukbonn.de (M.S.); stefan.kreyer@ukbonn.de (S.K.); jens.poth@ukbonn.de (J.M.P.); christian.putensen@ukbonn.de (C.P.);; 2 Department of Anesthesiology, Intensive Care and Pain Medicine, University Hospital Rostock, 18057 Rostock, Germany; jens-christian.schewe@med.uni-rostock.de

**Keywords:** COVID-19, ARDS, VV ECMO, interhospital transfer, primary ECMO transport, mortality

## Abstract

(1) **Background:** The COVID-19 pandemic presented significant challenges in managing acute respiratory distress syndrome (ARDS), with extracorporeal membrane oxygenation (ECMO) being a critical but resource-intensive intervention. (2) **Methods:** This retrospective study analyzed veno-venous (VV) ECMO therapy in ARDS patients before and during the pandemic at a high-volume ECMO center in Germany. The study used a reduced ECMO team (one medical and one nursing specialist) to optimize patient care with limited resources, aiming to offer insights for future pandemic management. Data from 181 adult patients (age ≥ 18) with severe ARDS were analyzed: 57 pre-pandemic and 124 during the pandemic. (3) **Results:** Despite increased isolation measures during the pandemic (25% pre-COVID-19 vs. 79% during COVID-19, *p* < 0.0001), there was no significant change in transportation mode (ground vs. air) or ECMO implantation times at local hospitals. Similarly, time and distance for primary ECMO transport remained unchanged between the two periods. Complication rates related to ECMO circuit placement and prolonged transport were also insignificant across groups. However, ECMO therapy duration (median 12 days pre-COVID-19 vs. 19 days during COVID-19, *p* < 0.0001) and hospital stays (median 3 days pre-COVID-19 vs. 7 days during COVID-19, *p* < 0.01) were longer during the pandemic. Mortality rates were also higher during the pandemic (49% pre-COVID-19 vs. 65% during COVID-19, *p* < 0.05). (4) **Conclusions:** In conclusion, a reduced ECMO team proved to be an effective resource-saving strategy that maintained high-quality care with low complication rates, despite the additional challenges posed by pandemic-related isolation measures.

## 1. Introduction

The COVID-19 pandemic has posed significant challenges for the global healthcare system, particularly in maintaining high-level care in intensive care units [1]. The surge in COVID-19 patients requiring intensive care has led to a shortage of both human and material resources [2,3]. COVID-19 disease can present as acute respiratory distress syndrome (ARDS), a life-threatening condition [4,5]. In cases of severe ARDS where conventional lung-protective mechanical ventilation fails to maintain adequate gas exchange, extracorporeal membrane oxygenation (ECMO) can be a life-saving intervention [6,7]. If a hospital lacks the necessary resources, technical capabilities, and expertise to perform ECMO, it may be crucial to deploy a specialized ECMO team from an ARDS/ECMO center to the peripheral hospital to perform the procedure and transport the patient back to the center for further care [8,9,10,11].

The use of ECMO during past pandemics, such as the 2009 H1N1 influenza pandemic, has proven successful when executed by a qualified ECMO team, including its preparation, implementation, and monitoring [8,9,12,13]. Similarly, early initiation of ECMO can be beneficial in managing COVID-19-related ARDS, as demonstrated by a multicenter trial which shows improved outcomes with timely ECMO intervention [14,15]. The Extracorporeal Life Support Organization (ELSO) registry reported a cumulative incidence of in-hospital mortality of approximately 37.4% for patients receiving VV ECMO for COVID-19, which is comparable to historical data from non-COVID-19 ARDS patients [16]. These data suggest that while ECMO can be effective, the mortality rate remains significant, underscoring the need for careful patient selection and timing of this specific intervention. Resource constraints during the pandemic have complicated the deployment of ECMO in patients with COVID-19-related ARDS. The high demand for intensive care resources, including VV ECMO, has led to ethical dilemmas regarding patient prioritization and the allocation of limited healthcare resources [17]. In some regions, the capacity to provide ECMO was strained, prompting discussions about the feasibility of transferring patients to facilities that were equipped for such specific interventions [18]. Furthermore, the prolonged use of VV ECMO in COVID-19 patients has been documented, with some cases requiring support for over 100 days. This raised concerns about the associated risks of complications and resource utilization [19].Taken together, VV ECMO has proven to be a vital component in the management of severe COVID-19 ARDS, with its efficacy being influenced by timely intervention, patient-specific factors, and resource availability. Because of the overload of local resources, patient transfer to a specialized center might be necessary. In such cases, however, a transfer to a secondary hospital may be possible only under ECMO support due to the severity of the disease and also because of the complications of the accompanying sepsis or septic shock [20]. This may potentially necessitate “primary ECMO”, even if it would not be indicated/necessary under normal circumstances [1,2,3,16,21].

Primary ECMO transport is a complex interhospital transfer that may require a significant number of medical personnel (ECMO team lead, cannulating provider, ECMO provider, ECMO specialist, medical transport team) and resources [9]. These transfers are even more challenging during a pandemic, owing to reduced resources and enhanced isolation measures. Studies have shown that ECMO interhospital transport is associated with additional challenges during pandemics [8,9,10,22,23,24,25,26]. Wearing personal protective equipment (PPE) and placing patients in isolation rooms are necessary measures to prevent the spread of infection. However, these measures can lead to complications in patients on ECMO. The additional time required to put on the protective equipment and establish the necessary isolation measures may delay the setting up of the ECMO procedure. Increased protective measures during ECMO can potentially worsen patient outcomes and hinder communication within an ECMO team. Additionally, the primary ECMO transport of patients back to a specialized ARDS/ECMO center may pose risks [8,9,10,22,23,24,25,26]. Transporting patients while maintaining isolation measures requires meticulous planning and coordination [22,25,26]. The increased burden on the ECMO team owing to the use of protective clothing can increase the complexity of transportation, jeopardizing patient safety [22,26]. During the early stages of the COVID-19 pandemic, many medical aspects were unclear, and strategic transfers were often uncoordinated due to capacity constraints.

Identifying best practices, minimizing risks, and optimizing the treatment course is essential to ensure the best possible care for patients, especially under the difficult conditions of a pandemic.

In this study, we compared data pertaining to ECMO implementation, complications, and outcome parameters (time on ventilation and length of hospital stay) before and during the COVID-19 pandemic in a high-volume ARDS/ECMO center in Germany. Here, a specialized reduced ECMO team approach, which includes one medical and one nursing specialist, has been established for years. The following analysis examined the use of this specialized reduced ECMO team approach in conjunction with the increased isolation measures mandated during the COVID-19 pandemic [8]. The objective was to investigate whether the enhanced isolation measures due to the COVID-19 pandemic had an impact on patient safety, medical outcomes, duration of interhospital transport, and mortality when a resource-saving reduced ECMO team approach was utilized. Insights gained from this study can help shape the future of intensive medical care for severe ARDS during future pandemics. Moreover, it may help optimize the safety and effectiveness of this resource-sparing primary ECMO transport approach and provide a basis for planning for special and high-risk interhospital transports and possible strategic intensive care transfers.

## 2. Materials and Methods

### 2.1. Study Design

This was a retrospective single-center comparative observational study. The study protocol complied with the principles enshrined in the Declaration of Helsinki and the “Strengthening the Reporting of Observational Studies in Epidemiology (STROBE)” recommendations.

### 2.2. Ethics

This study was approved by the ethics committee of the University Hospital Bonn (no. 042/21). Patient consent was waived because of the retrospective study design.

### 2.3. Study Variables

Data pertaining to the following variables were recorded: number of ECMO team deployments; isolation status according to the hygiene guidelines of the Robert Koch Institute, Germany [27]; mode of transportation, ECMO implementation on-site, duration of ECMO cannulation and length of stay in the external hospital, transport duration, and route of the primary ECMO transport; complications and their weighting; treatment-related aspects (treatment duration, ventilation time, and duration and outcome of ECMO therapy).

### 2.4. Study Population

Data were collected from all veno-venous (VV) ECMO patients treated between March 2018 and February 2022 in the intensive care units of the Department of Anesthesiology and Intensive Care Medicine at the University Hospital Bonn. 

Inclusion criteria were as follows:

(1) age ≥ 18 years;
(2) availability of medical records, including VV ECMO treatment data, transport protocols, and electronic patient records including vital parameters and blood gas analyses;
(3) ARDS according to the Berlin definition [28];
(4) SARS-CoV-2 PCR testing.

Exclusion criteria:

Patients who received veno-arterial ECMO (VA ECMO) for isolated cardiac support without ARDS were excluded. These patients were transported by our ECMO team, but the focus of the present manuscript is on the impact of the pandemic on isolation measures and their role in the potential complication of primary VV ECMO implantations. The VA ECMO patients did not suffer from COVID-19-related ARDS, so no reasonable comparison between VA-ECMO runs before COVID-19 and during the COVID-19 pandemic was possible. Moreover, VA ECMO patients undergo a different cannulation approach and thus have a different accompanying risk profile.

### 2.5. Indication for VV ECMO

The indications for VV ECMO support were in accordance with the Extracorporeal Life Support Organization (ELSO) guidelines, such as the treatment of severe hypoxemia and hypercapnia and/or avoidance of potentially harmful mechanical ventilation (i.e., prolonged use of excessively high peak inspiratory pressures or a pressure difference of >15 cm H_2_O) after exhausting all conservative treatment options to ensure adequate gas exchange according to ARDS network definitions [29].

### 2.6. ECMO Team and Cannulation Strategy

The ECMO team of the University Hospital Bonn consists of ECMO physicians (anesthesiology specialists with additional qualifications in intensive care medicine) and ECMO intensive care nurses (intensive care and anesthesia nurses with at least 3 years of experience in intensive care medicine and treatment of ARDS) [8]. In addition, all team members are trained in accordance with the requirements of the German Interdisciplinary Association for Intensive Care and Emergency Medicine (DIVI). A 24 h on-call service, available 365 days a year, consisting of an ECMO doctor and ECMO nurse, can be alerted at any time via a 24 h hotline. The team ensures on-site care for ECMO implantation in an external hospital and carries out the accompanying interhospital transport. This interprofessional ECMO team is deployed within 60 min. The decision on the ECMO indication and implementation of ECMO therapy was made by the ECMO team on-site. The interprofessional ECMO team performed all cannulation steps. After establishing ECMO and stabilizing the critically ill patient, safe interhospital transport to the ARDS/ECMO center is crucial. VV ECMO was established using an ultrasound-guided percutaneous puncture. Cannulas were implanted in either the two femoral veins (bifemoral cannulation approach) or via a femoral vein and the right jugular vein (femoro-jugular cannulation approach).

### 2.7. Definition of Complications

To assess the frequency of complications during primary ECMO transport, complication rates were categorized as follows:

- Complications related to ECMO implementation (e.g., difficult cannulation, multiple punctures of the respective vessel, repositioning, arterial malpuncture, immediate need for a 3rd cannula);
- Technical failure (e.g., failure of equipment of the ICU stretcher, ambulance, or helicopter [such as electrical outlets] or medical equipment such as syringe pumps, or monitoring devices]);
- Complications related to the ECMO system (e.g., console technology such as touchscreen, software, oxygenator/tube system (system pressure, air trapping, performance, etc.)).
- Medical failure (e.g., hemodynamic instability, respiratory problems, oxygen saturation, abdominal elevated pressure, or bleeding);
- Personnel-related complications collectively referred to as crew resource management (due to the difficult communication caused by the PPE and the spatial separation from the patient due to isolation measures).

### 2.8. Statistical Analyses

The normality of the distribution of continuous variables was assessed using the Shapiro–Wilks test. Non-normally distributed continuous variables are presented as median and interquartile range (IQR), while normally distributed continuous variables are presented as mean ± standard deviation. Categorical variables are presented as frequency (percentage). Between-group differences were assessed for statistical significance using the unpaired *t*-test (normally distributed variables), Wilcoxon test (nonnormally distributed variables), and Fisher-Yates test or Chi-square test (categorical variables). Microsoft Excel Version 16.88 (Microsoft Corporation, Redmond, WA, USA) was used to create the database. Statistical analyses were performed using R Studio version 1.4.1106 using R version 4.2. Statistical significance was set at *p* < 0.05.

Power analysis: no pre-study power analysis was performed. This is a retrospective data analysis of all available VV ECMO cases. After cessation of the COVID-19 pandemic, no further cases could be recruited. Hence, we analyzed the available cases and evaluated them regarding statistical significance based on identical timeframes comparing cases pre-COVID-19 pandemic versus COVID-19 pandemic.

Missing data: variables were included in the study and were analyzed when the fraction of missing data did not exceed more than 20%. Variables with a fraction of more than 20% would have been excluded (not applicable in our dataset).

Data sources: The patients’ individual electronic data were analyzed. This included the scanned, analog (non-electronic) ECMO mission protocols, from which insertion times, cannulation times, complications, and all other information related to ECMO implantation were extracted. In addition, our departmental ECMO database was used to determine the ECMO interventions performed annually and to identify cases before and during the COVID-19 pandemic.

## 3. Results

### 3.1. Characteristics of the Study Cohort

**Figure 1:**
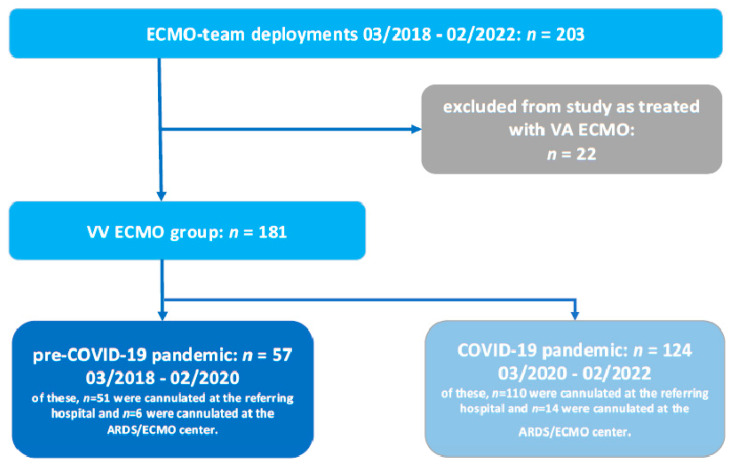
Schematic illustration of the selection of eligible study cohort of ECMO team deployments. ECMO: extracorporeal membrane oxygenation; VV veno-venous; VA: veno-arterial.

During the study reference period, 203 patients who were transported by the ECMO team were treated with ECMO (Figure 1). Out of these, 181 VV ECMO patients qualified by the inclusion criteria and were included in this study. The cohort was divided into a pre-COVID-19 pandemic group (*n* = 57; of these, 51 were cannulated at the referring hospital) and a COVID-19 pandemic group (n = 124; of these, 110 were cannulated at the referring hospital). 

**Figure 2:**
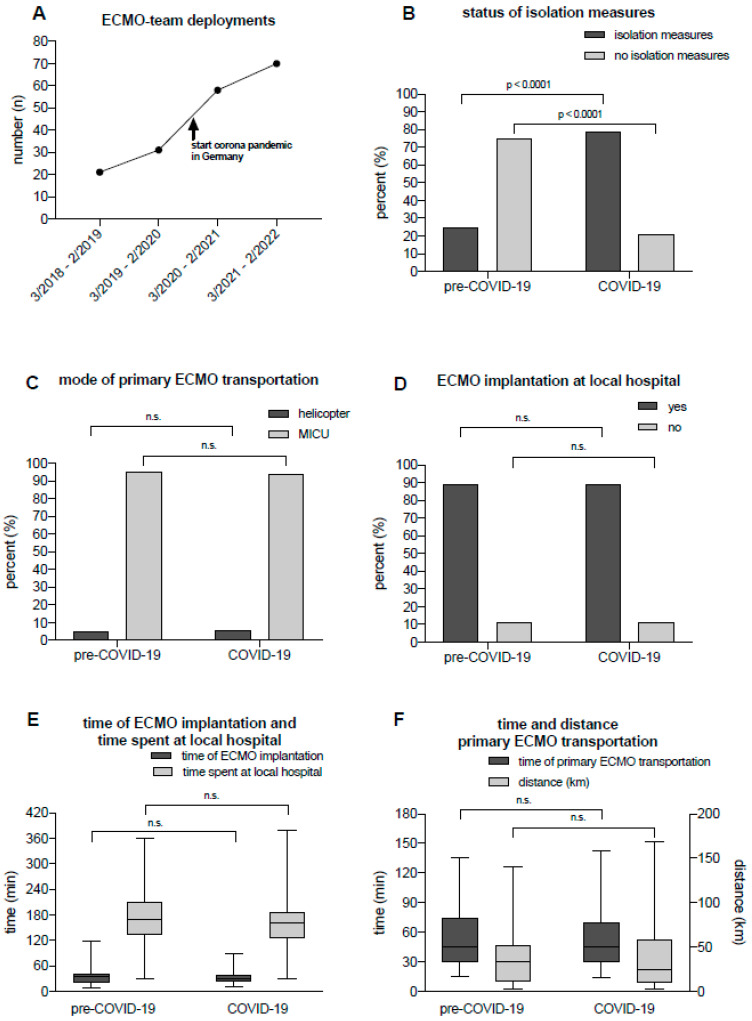
(**A**) Number of ECMO team deployments in the observation period before (pre-COVID-19) and during the pandemic (COVID-19). (**B**) Implementation of isolation measures (PPE use and isolation in appropriate rooms) before (pre-COVID-19) and during the pandemic (COVID-19). There was a significant increase in the implementation of isolation measures during the COVID-19 period (*p* < 0.0001 vs. pre-COVID-19, Chi-square test). (**C**) Mode of primary ECMO transportation back to the ECMO/ARDS center before (pre-COVID-19) and during the pandemic (COVID-19) (Chi-Square test, *p* = n.s.). (**D**) Frequency of on-site VV ECMO implantation at the local hospital prior to the start of primary ECMO transport before (pre-COVID-19) and during the pandemic (COVID-19) (Chi-Square test, *p* = n.s). (**E**) Time required for ECMO implantation and time spent at the local hospital before (pre-COVID-19) and during the pandemic (COVID-19) (Chi-Square test, *p* = n.s.). (**F**) Time and distance of primary ECMO transports before (pre-COVID-19) and during the pandemic (COVID-19) (Chi-Square test, *p* = n.s.). ECMO: extracorporeal membrane oxygenation; MICU: mobile intensive care unit; PPE: personal protective equipment; n.s.: not significant.

In Germany, the COVID-19 pandemic started at the end of February 2020. Therefore, the COVID-19 era was defined as the two-year interval from March 2020 to February 2022. The pre-COVID-19 era, as a control group, was defined as the two-year interval from March 2018 to February 2020 (Figure 2A).

Demographic data are summarized in Supplemental Table S1.

### 3.2. No Delay in Medical Procedures during ECMO Team Deployment

To determine whether the use of a specialized reduced ECMO team approach in conjunction with the increased isolation measures mandated during the COVID-19 pandemic would have an impact on the medical procedures, we analyzed the number of ECMO team deployments, the status of isolation measures, the mode of primary ECMO transportation, and whether the ECMO implantation happened at the local hospital. In addition, we investigated the time spent at the local hospital, the time to perform the ECMO implantation, and the time and distance of the primary ECMO transport.

The number of transports increased over the observation period; an increase in interhospital transport was particularly evident during the COVID-19 pandemic (Figure 2A). The proportion of patients requiring isolation measures increased significantly during the COVID-19 pandemic (pre-COVID-19: 14/57 [25%] vs. COVID-19: 98/124 [79%], *p* < 0.0001, Figure 2B). Ground-based transportation was almost exclusively the primary means of ECMO transport to the ARDS/ECMO center in both groups (Figure 2C, *p* = not significant [n.s.]). Both cohorts had an equal frequency of on-site establishment of VV ECMO (89%) and transport of patients with established ECMO therapy (Figure 2D, *p* = n.s.). In other cases, optimized therapy ensured sufficient gas exchange, or the ECMO team deemed that the patient could be safely transported without established ECMO therapy. There was no significant between-group difference with respect to the duration of the ECMO cannulation procedure (time from the start of ECMO cannula implantation to the start of extracorporeal circulation) (Figure 2E, *p* = n.s.). The total time spent on-site by the ECMO retrieval team in the external hospital also did not change under additional protective isolation measures and their potential consequences (Figure 2E, *p* = n.s.). There were also no significant between-group differences regarding the transport duration (defined as time from the start of the primary ECMO transport at the referring hospital until arrival at the ECMO/ARDS center) and distance of the primary ECMO transport (Figure 2F, *p* = n.s.).

These results show that the increased number of ECMO team deployments and the elevated isolation measures did not lead to a delay in medical procedures during the mission of the ECMO team.

### 3.3. No Increased Complication Rate during ECMO Insertion and Transport

Increased isolation measures during the ECMO team deployment could be a risk factor for an elevated rate of complications. We therefore investigated whether significant complications occurred during the EECMO implementation and listed them in five categories (for a detailed definition of the various complications please refer to Section 2.7 in Section 2).

**Figure 3:**
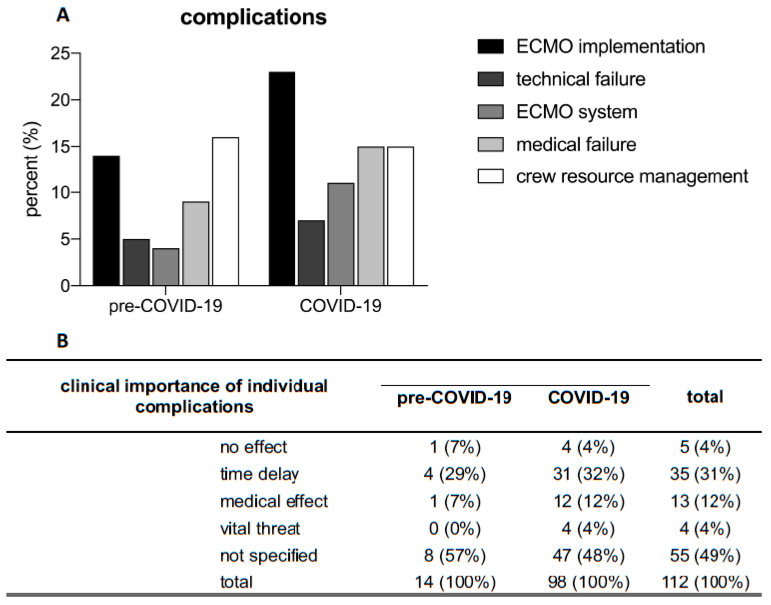
(**A**) Frequency of complications, categorized into those related to ECMO implementation (e.g., difficult cannulation, multiple punctures of the respective vessel, repositioning, arterial malpuncture, immediate need for a 3rd cannula), technical failure (e.g., failure of equipment of the ICU stretcher, ambulance or helicopter [such as electrical outlets] or medical equipment [such as syringe pumps, monitoring devices]), ECMO system (e.g., console technology such as touchscreen, software, oxygenator/tube system [system pressure, air trapping, performance, etc.]), medical failure (e.g., hemodynamic instability, respiratory problems, oxygen saturation, abdominal elevated pressure, or bleeding), and personnel-related complications collectively referred to as crew resource management (caused by communication barriers due to the use of PPE and the spatial separation from the patient). There was no significant difference between the COVID-19 period and the pre-COVID-19 period with respect to complication rates in any of the five categories (Chi-Square-test, *p* = n.s.). (**B**) Clinical importance of individual complications. No significant differences were observed between the two cohorts (Chi-square test, *p* = n.s.). ECMO: extracorporeal membrane oxygenation; ICU: intensive care unit; PPE: personal protective equipment; n.s.: not significant.

There was no significant increase in complications associated with ECMO implementation due to enhanced isolation measures (Figure 3A, *p* = n.s.). There were no significant differences regarding the complication rates in the COVID-19 pandemic compared to the pre-COVID-19 pandemic in all five categories listed, despite the challenge posed by enhanced increased isolation measures and their direct consequences (Figure 3A, *p* = n.s.). The assessment of these complications performed by the ECMO teams immediately after the operation also showed no differences between the two cohorts (Figure 3B, *p* = n.s.).

These findings demonstrate that no increase in complications was observed during ECMO insertion and transport despite the burden of increased isolation measures.

### 3.4. Outcome

To investigate whether the COVID-19-related ARDS outcome varies when compared with the ARDS of the pre-COVID-19 era, we analyzed the time of treatment, the RESP, SOFA, and CCI score as well as the in-hospital mortality.

**Figure 4:**
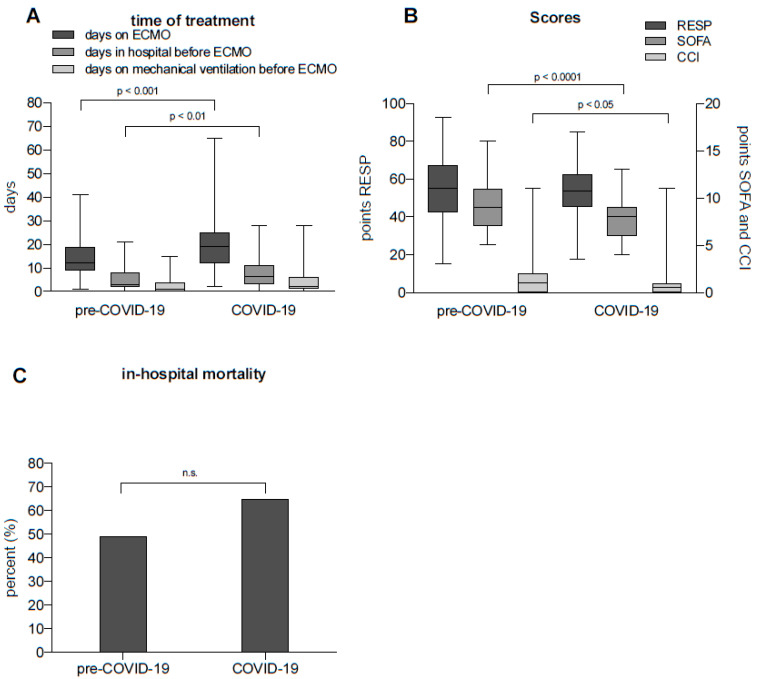
(**A**) Number of days patients spent on ECMO, duration of hospitalization, and number of days on mechanical ventilation before ECMO implantation, before (pre-COVID-19) and during the pandemic (COVID-19). Pre-COVID-19: days on ECMO vs. COVID-19: days on ECMO, Wilcoxon rank sum test, *p* < 0.001. Pre-COVID-19: days in hospital before ECMO vs. COVID-19: days in hospital before ECMO, Wilcoxon rank sum test, *p* < 0.01. (**B**) Comparison of RESP scores, SOFA scores, and CCI before (pre-COVID-19) and during the pandemic (COVID-19). Pre-COVID-19: SOFA vs. COVID-19: SOFA, Wilcoxon rank sum test, *p* < 0.0001. Pre-COVID-19: CCI vs. COVID-19: SOFA, Wilcoxon rank sum test, *p* < 0.05. (**C**) The in-hospital mortality rate during the pandemic (COVID-19) was significantly higher than that during the pre-COVID-19 period (Chi-square test, *p* < 0.05). ECMO: extracorporeal membrane oxygenation; RESP: Respiratory ECMO Survival Prediction Score; SOFA: Sequential Organ Failure Assessment Score; CCI: Charlson Comorbidity Index; n.s.: not significant.

In patients with severe ARDS, the duration of ECMO therapy was longer during the COVID-19 pandemic than during the pre-COVID-19 pandemic period (pre-COVID-19: median12 [IQR 10–19] days vs. COVID-19: median 19 [IQR 12–25] days, *p* < 0.0001, Figure 4A). During the COVID-19 pandemic, the duration of previous hospitalization for VV ECMO was significantly longer than during the pre-COVID-19 pandemic period (pre-COVID-19: median 3 [IQR 2–8] days vs. COVID-19: median 7 [IQR 3–11] days, *p* < 0.01, Figure 4A). However, the number of days on mechanical ventilation did not differ between the two observation periods (Figure 4A, *p* = n.s.). There was no significant difference between the two periods with respect to the Respiratory ECMO Survival Prediction Score (RESP) (Figure 4B, *p* = n.s.). However, the Sequential Organ Failure Assessment Score (SOFA) and Charlson Comorbidity Index (CCI) were lower in the COVID-19 pandemic period than in the pre-COVID-19 pandemic period (Figure 4B, *p* < 0.0001 and *p* < 0.05, respectively). The number of patients that were treated with ECMO and died was significantly higher during the COVID-19 pandemic (pre-COVID-19: deceased 28/57 (49%) vs. COVID-19: 80/124 (65%), *p* < 0.05, Figure 4C).

These results show a difference in various outcomes between the COVID-19 era and the pre-COVID era. 

## 4. Discussion

This retrospective data analysis aimed to draw lessons from the COVID-19 pandemic regarding the primary ECMO interhospital transport of patients with severe ARDS. The insights gained should enable better material and personnel resource planning for possible future pandemics, in line with the guiding principle of Fit-for-Future. Our data show that despite the increase in primary ECMO team deployments and tripling of isolation measures during the COVID-19 pandemic, the complication rate associated with ECMO placement and primary ECMO transport remained unchanged when performed by a specialized team with extensive expertise. Furthermore, there was no extension of team deployment time at the external hospital or transport time. However, during the COVID-19 pandemic, patients with severe ARDS required more treatment days on ECMO and had a higher mortality rate compared to those in the pre-COVID-19 pandemic period.

The first lesson learned from our study is that our approach of reducing the number of ECMO team members, which was already considered in the ELSO guidelines, is effective even under pandemic conditions [8,9]. This was likely attributable to the high level of experience within individual ECMO teams and extensive training of the ECMO team in the standard operating procedures. These factors enabled the ECMO team to handle a significantly higher volume and complexity of operations with the same personnel. This established routine contributed to the fact that the deployment times at external hospitals and the duration of the primary ECMO transport remained unchanged compared to the pre-COVID-19 pandemic period. Others studies reported similar data during the H1N1 pandemic when a highly trained ECMO team performed the ECMO preparation and implementation [8,9,12,13]. Moreover, we believe that our data further supports the hypothesis that the deployment of a reduced ECMO team approach is feasible even in a pandemic scenario. This is of importance, as recent studies suggest an early initiation of VV ECMO in managing COVID-19 related ARDS to improve outcome [14,15]. Thus, a reduced ECMO team approach might help to maintain VV-ECMO as a timely treatment option for ARDS patients despite a pandemic scenario with limited resource availability. 

The second lesson from our study is that despite the increased volume of operations and challenging circumstances during the COVID-19 pandemic, our ECMO team approach maintained a consistent safety profile, with no rise in complications associated with this highly complex therapy and interhospital transport. This is particularly noteworthy, as interhospital transport, especially primary ECMO transport, is a high-risk activity that can compromise patient safety [22,24,25,26,30]. There is a growing consensus on the need for ECMO teams to adopt standardized workflows, regular training, a uniform definition of complications (which must also be recorded and followed up in a structured manner), and a high volume of ECMO operations to minimize the rate of complications [9,24,25]. While our study found no difference in the immediate complication rate of primary ECMO transports, it revealed an increased in-hospital mortality during the COVID-19 pandemic. One potential explanation is that transporting these patients during the pandemic led to increased mortality, even if there was no difference in the immediate complication rate. More likely, COVID-19-associated ARDS has a worse mortality rate than most other conditions treated with VV ECMO [16].

The third key takeaway from our data are that transportation times for ambulances carrying the ECMO team to external hospitals and for primary ECMO transport back to the ARDS/ECMO center did not increase during the COVID-19 pandemic. This suggests that the pandemic did not negatively impact other rescue service aspects. The interhospital transport concept of the Emergency Medical Services of Bonn has a special feature whereby any rescue vehicle (ambulance of the primary rescue service) can be used for specialized interhospital transport. All vehicles are equipped to accommodate intensive care transport, with a special intensive care transport stretcher replacing the regular rescue stretcher, as needed. Additional equipment, such as syringe pumps, ventilator, oxygen cylinders, and the ECMO console, is securely loaded onto this stretcher using a custom holder. This allows the ECMO team and its specialized equipment (cannula bag, HLS set bag, ECMO console, tube heater, etc.) to be rapidly transported to external hospitals and back to an ARDS/ECMO center 24/7. In this concept, any ambulance can be dispatched for primary ECMO transport, and is instantly converted to a mobile intensive care unit (MICU) by swapping the regular stretcher with a special intensive care transport stretcher. This approach offers a cost-efficient, straightforward, and rapidly available solution for time-critical primary ECMO operations. In contrast, other transport concepts in other rescue service areas rely on specially equipped vehicles, which may not be available round-the-clock [3]. If these vehicles are already committed to another transport assignment (often for several hours), they become unavailable for ECMO operations.

The insights from this study can have a positive impact during a pandemic when resources are scarce or insufficient. Strategies that can conserve resources (both materials and personnel) are crucial during a pandemic. 

The authors of this retrospective analysis conclude that a reduced ECMO team approach might be a suitable option to provide a constantly available 24/7 ECMO service despite potentially limited resource availability. In this case, to save resources, the reduced ECMO team consists of only one medical and one nursing specialist. Our data show, albeit with certain limitations due to the retrospective nature of the analysis, that this reduced ECMO team concept can cope with an increased mission volume, even if increased isolation measures are required. We are convinced that it is advantageous for the planning and execution of such highly complex interhospital transport if a suitable means of transportation can be provided immediately by the local EMS. It is also extremely important that the EMS personnel involved are appropriately trained in the planning, execution, and application of ECMO. Therefore, the above-mentioned interhospital transport concept used in this study might help to better manage limited resources in the event of a pandemic.

A key limitation of this study is the retrospective observational design and monocentric nature of the analyses. However, randomized controlled studies on this topic will be very difficult to realize for multiple reasons, including the fact that the current pandemic is now over. In addition, our results are based on existing routine documentation, which may have introduced an element of selection bias because we are unaware what may have happened with other patients. Furthermore, the design of this retrospective monocentric study is also a limiting factor, as it was only matched for “time period” without the possibility of a power analysis. Lastly, with our long-standing expertise as a high-volume ARDS/ECMO center, we are comfortable and well versed with the reduced ECMO team approach. On the other hand, while the reduced ECMO team approach may offer staffing advantages for smaller centers, it also has the disadvantage of requiring potentially less experienced staff to perform many more tasks from a single person.

In summary, this study demonstrates that a reduced ECMO team approach during a pandemic can achieve resource-efficient use of materials and personnel while maintaining quality and consistently low complication rates, despite the additional isolation measures.

## Data Availability

The clinical datasets generated and/or analyzed during the current study are not publicly available due to the local data protection law but are available from the corresponding author on reasonable request.

## Supplementary Materials

The following supporting information can be downloaded at: https://www.mdpi.com/article/10.3390/jcm13185391/s1, Table S1: Demographic data.
