# Supplementary material for: Fit-for-Future: Lessons Learned from the COVID-19 Pandemic in Primary Extracorporeal Membrane Oxygenation (ECMO) Transports of Acute Respiratory Distress Syndrome (ARDS) Patients
Source: J Clin Med. 2024 Sep 12;13(18):5391. doi: 10.3390/jcm13185391 (PMC11432369; doi:10.3390/jcm13185391)
Supplement: Supplementary file 1 [file jcm-13-05391-s001.zip › jcm-3178332-supplementary.pdf]

Supplemental Table S1

Characteristics	Variable	pre-COVID-19	COVID-19	Total	Test
Sex	Male	39 (68%)	91 (73%)	130 (72%)	p-value: 0.4903 (Pearson's Chi-squared test)
	female	18 (32%)	33 (27%)	51 (28%)	
	total	57 (31%)	124 (69%)	181 (100%)	
Age [years]	min/max	27/80	21/77	21/80	p-value: 0.6303 (Wilcoxon rank sum test)
	median [IQR]	58 [46–65]	57 [49–62]	57 [48–63]	
Height [cm]	min/max	149/195	150/196	149/196	p-value: 0.9119 (Wilcoxon rank sum test)
	median [IQR]	175 [170–185]	175 [170—180]	175 [170–180]	
weight [kg]	min/max	50/260	60/180	50/260	p-value: 0.7789 (Wilcoxon rank sum test)
	median [IQR]	90 [80–110]	94 [85–110]	93 [85–110]	
Body mass index	min/max	18/95	21/54	18/95	p-value: 0.6250 (Wilcoxon rank sum test)
	median [IQR]	29 [26–38]	30 [28–36]	31 [27–36]	
